# Effects of thoracic manipulation with trigger point therapy on inflammatory cytokine levels in individuals with multiple sclerosis: a pilot study

**DOI:** 10.3389/fresc.2026.1699274

**Published:** 2026-04-08

**Authors:** Murdi S. Alanazi, Robert W. Motl, Benjamin A. Jones, Haiyan Qu, Peng Li, William R. Reed

**Affiliations:** 1 Department of Physical Therapy and Health Rehabilitation, College of Applied Medical Sciences, Jouf University, Sakaka, Saudi Arabia; 2 Rehabilitation Science Program, University of Alabama at Birmingham, Birmingham, AL, United States; 3 Department of Kinesiology and Nutrition, College of Applied Health Sciences, University of Illinois Chicago, Chicago, IL, United States; 4 Division of Neuroimmunology and Multiple Sclerosis, Department of Neurology, Heersink School of Medicine, University of Alabama at Birmingham, Birmingham, AL, United States; 5 Department of Health Services Administration, School of Health Professions, University of Alabama at Birmingham, Birmingham, AL, United States; 6 School of Nursing, University of Alabama at Birmingham, Birmingham, AL, United States; 7 Department of Physical Therapy, School of Health Professions, University of Alabama at Birmingham, Birmingham, AL, United States

**Keywords:** biomarkers, chiropractic, cytokines, inflammation, manual therapy, multiple sclerosis, serum, spinal manipulation

## Abstract

**Background:**

Spinal manipulation (SM) may modulate immune and inflammatory responses in healthy and/or musculoskeletal pain populations, yet SM responses in neurodegenerative populations such as multiple sclerosis are essentially unknown. This pilot study estimated the potential effects of chiropractic thoracic SM combined with trigger point therapy on serum inflammatory cytokine/chemokine levels, neurodegeneration biomarkers, and clinical/performance-based outcomes in people with relapsing-remitting multiple sclerosis (RRMS). The goal was to inform the design of future research.

**Methods:**

This pilot randomized, sham-controlled trial included 21 RRMS participants assigned to either SM (*n* = 11) or sham-SM (*n* = 10) groups. Interventions were delivered twice weekly for four weeks. Blood samples were collected at five timepoints: baseline (T0), 20 min and 2 h after first intervention (T1 and T2 respectively), and 20 min and 2 h after the final intervention (T3 and T4 respectively). Overall 21 inflammatory biomarkers, 3 neurodegenerative biomarkers and 12 clinical/performance outcomes were assessed. Between-group differences were evaluated by comparing change scores from baseline per group, and effect sizes were reported using Cohen's *d*.

**Results:**

Eight cytokines/chemokines in the SM group demonstrated moderate to large effect sizes (d ≥ 0.5) at a single timepoint post-intervention compared with the sham-SM group, whereas six (IL-8, IL-17A, GM-CSF, MIP-1β, IFN*γ*, Fractalkine) demonstrated moderate to large effect sizes at multiple timepoints post-intervention. Among neurodegeneration biomarkers, t-tau levels decreased in the SM group with a small effect size (d = −0.42). Most clinical- and performance-based outcomes had small effect sizes with the few moderate effect size changes being below clinical relevance thresholds.

**Conclusion:**

This study identified six cytokines/chemokines that had moderate to large effect sizes at multiple post-intervention timepoints favoring SM. Of these biomarkers, all are considered to be primarily pro-inflammatory. Such results support appropriately powered randomized controlled trials of SM in RRMS population that focus on evaluating these cytokines/chemokines across multiple timepoints including immediately (< 5 min), intermediately (< 30 min), and short-duration (≥2 h) post-intervention and seek to determine the contribution of soft tissue stimulation (i.e., trigger point therapy) preceding the SM to cytokine/chemokine response.

**Clinical Trial Registration:**

https://clinicaltrials.gov/ NCT04972929, registration date: April 27, 2021.

## 1 Introduction

Multiple sclerosis (MS) is a chronic neurological disease characterized by inflammation, demyelination, and neuroaxonal loss within the central nervous system (CNS) (1). The pathogenesis of MS involves the infiltration of peripherally activated leukocytes, primarily T cells, across the blood-brain barrier into the CNS (2). This infiltration triggers the recruitment of inflammatory immune cells, which subsequently release a variety of inflammatory cytokines involved in demyelination and axonal injury (3). This pathogenic expression is typically seen in the 85% of people with MS who present with Relapsing Remitting MS (RRMS) disease, and marked by partial or complete recovery from acute exacerbations (4). Over time, people with RRMS frequently experience a wide range of debilitating symptoms, including fatigue, pain, cognitive impairment, depression, and motor dysfunction, which significantly impact quality of life (5–7).

Inflammatory cytokines are small signaling proteins produced by numerous cells (macrophages, monocytes, T helper cells, nonimmune nucleated cells, etc.) that play a central role in the pathophysiology of MS by regulating immune and inflammatory responses via serving as chemotactic factors, cell-to-cell communication, and immune cell differentiation regulators. Certain inflammatory cytokines such as interleukin-1beta (IL-1β), interleukin-17A (IL-17A), and tumor necrosis factor alpha (TNF-α) are classified as pro-inflammatory mediators, while others such as interleukin-4 (IL-4) and interleukin-10 (IL-10) are associated with anti-inflammatory functions. However, cytokines such as interleukin-2 (IL-2), interleukin-6 (IL-6), and interleukin-21 (IL-21) exhibit both pro- and anti-inflammatory properties but tend to promote pro-inflammatory responses in autoimmune diseases (8–10). A 2019 systematic review analyzing 226 studies showed that twenty-one blood cytokines and thirteen cerebrospinal fluid (CSF) cytokines were significantly elevated in MS patients compared to control (11). Newer studies support and expand these review findings demonstrating increased and/or decreased levels of both pro-inflammatory and anti-inflammatory cytokines in people with MS (12, 13). Elevated levels of pro-inflammatory cytokines, such as IL-2, have been reported during the relapsing phase of RRMS (14), whereas increased levels of anti-inflammatory cytokines, including IL-4 and IL-10, are often observed during the remission phase (15, 16). Moreover, elevated levels of pro-inflammatory cytokines, such as IL-6 and TNF-α, have been associated with the disease severity (17, 18), while reduced levels of anti-inflammatory cytokine IL-10 have been observed in the progressive forms of the disease (19). Together, these findings highlight the interplay role of inflammatory cytokines in the MS population which warrant further investigation into pharmacological and non-pharmacological interventions that can potentially modify these pro- and anti-inflammatory cytokines.

In addition to pro- and anti-inflammatory cytokines, astrocytic activity and neural damage biomarkers such as glial fibrillary acidic protein (GFAP), neurofilament light (NfL), and total tau (t-tau) are emerging as valuable biomarkers in MS pathophysiology, reflecting disease progression, and/or response to intervention strategies (20–24). These proteins are released into the extracellular space following astrocytic or neuronal injury, and their concentrations can be detected in CSF, plasma, and/or serum (24–30). Elevated levels of GFAP, NfL, and t-tau have been reported in individuals with MS compared to healthy controls and have been associated with MS disease severity, neurodegenerative activity, and imaging findings (25, 27, 29, 31).

Spinal manipulation (SM) is among the non-pharmacological manual therapy techniques commonly used by different healthcare professions such as chiropractic, osteopathy, and physical therapy. SM typically involves the application of a passive, high-velocity, low-amplitude thrusts into a spinal joint complex within its anatomical limits, with the goal of restoring optimal joint motion and function, and/or reducing musculoskeletal pain (32). Although the underlying mechanisms of SM and manual therapy as a whole are not yet fully understood, there is some evidence suggesting that SM exerts neuroimmune effects, modulating the immune response by decreasing pro-inflammatory cytokines and increasing anti-inflammatory cytokines (33–35). A 2021 systematic review of 13 studies evaluating the relationship between SM and immune system outcomes reported that SM was possibly associated with short-term changes in select immunological biomarkers including IL-1β, IL-2, and TNF*α* among asymptomatic participants or patients with low back pain, but the clinical relevance of these findings and the duration of these changes remain unknown as biomarkers of interest were assessed *in vitro* in a majority of these SM studies (36). For example, in healthy individuals who received a single thoracic SM, reduced *in-vitro* synthesis of TNF-α and IL-1β, and elevated IL-2 levels at 20 min and 2 h post-intervention were reported (37, 38). In individuals with acute and chronic low back pain (LBP), six lumbar SM sessions delivered over two weeks significantly decreased *in-vitro* production of TNF-α, IL-6, soluble tumor necrosis factor receptor 2 (sTNFR2), and interferon gamma (IFN-*γ*) in the chronic LBP group, whereas only TNF-α was reduced in the acute LBP group (39). Additionally in patients with LBP, eight sessions of instrument-assisted lumbar SM was associated with a reduction in urinary TNF-α levels (40). Conversely, findings from other SM and manual therapy studies reported little to no change in various immunological biomarkers (34, 41, 42). For instance, a study involving individuals with neck pain who received a single combined intervention (spinal mobilization and manipulation) reported no significant changes in cytokine levels at 10 min or 2 h post-intervention compared to controls, despite comprehensive analysis of both *in-vitro* stimulated and serum cytokines (43). Multiple systematic reviews report that there is currently insufficient evidence to draw conclusions as to the effect of SM or other manual therapies on cytokine profiles with one review specifically noting that it would be premature to conduct powered randomized clinical trials without additional evidence from exploratory clinical studies in healthy and pathological clinical populations (34, 36, 41).

Given the potential for SM to affect neuroimmune responses, this pilot randomized controlled trial examined the impact of a single and a series of 8 chiropractic thoracic SM with trigger point therapy on immunological biomarkers at 20 min and 2 h post-intervention along with effects on select clinical- and performance-based outcomes in people with RRMS to inform future fully powered clinical trials. We examined the short, intermediate, and cumulative effects of thoracic SM compared with a sham-SM intervention on 21 serum cytokine/chemokine concentrations, neuroaxonal biomarkers (GFAP, NfL, t-tau), and MS-related symptoms including fatigue, pain, depression, sleep disturbance, motor function, and cognitive processing speed. Such evidence is important to characterize with the goal of informing future SM-related trials in neurodegenerative populations.

## 2 Materials and methods

### 2.1 Study design

This study was a pilot parallel-group, randomized sham-controlled trial registered with clinical trials.gov (NCT04972929) and conducted at the University of Alabama at Birmingham (UAB). A parallel-group design was preferred to a crossover design due to potential biomarker carryover effects, as well as the increased number of total office visits that would be involved. Ethical approval was obtained from the UAB Institutional Review Board prior to participant enrollment with all participants providing written informed consent.

### 2.2 Participants

People with RRMS were recruited through advertisements distributed on the UAB campus, and the local chapter of the National Multiple Sclerosis Society as well as working with clinicians at the UAB Multiple Sclerosis Center. Inclusion criteria consisted of: (1) age 18–55 years; (2) physician-confirmed diagnosis of RRMS; (3) Expanded Disability Status Scale (EDSS) score below 4.5 (fully ambulatory without aid); (4) relapse-free in the last 30 days; (5) no known cardiovascular, pulmonary, or metabolic disease; (6) currently on stable FDA-approved MS disease-modifying therapy; (7) no contraindications to thoracic spinal manipulation (i.e., thoracic spine fracture, spinal infection, malignancy/cancer, osteoporosis); and (8) acceptance of informed consent. Exclusion criteria included: (1) uncontrolled hypertension (systolic >160 mmHg, diastolic >95 mmHg); (2) history of spinal surgery or recent history of bone fractures; and (3) pregnancy in the last 12 months. A detailed medical history of medication usage, disease modifying therapy regimen, and/or corticosteroid exposure was not taken prior to study enrollment. The first participant was enrolled in the study on September 15, 2023.

### 2.3 Randomization, blinding, and sample size

Participants meeting the inclusion criteria were randomized using simple randomization via a random number generator generating odd/even whole integers into either the thoracic SM group or the thoracic sham-SM group (Microsoft software, Microsoft Redmond WA). Participants, outcome assessors, and data analysts were blinded regarding allocation. Due to the nature of the manual therapy intervention, the chiropractic clinician administering the intervention/sham intervention was not blinded, but was uninvolved in outcome assessments. Participants were randomly allocated at a 1:1 ratio into the thoracic SM or sham-SM groups. The target sample of 12 participants per group was estimated based on previous studies (44) and recommendations of 12 participants per group for obtaining reliable estimates in a pilot study with the goal for the planning of larger trials (45).

### 2.4 Procedures

Participants were initially screened via a telephone conversation with the study coordinator and if the inclusion criteria were met, individuals were invited to attend an in-person informational session in which the study was explained, informed consent obtained, and thoracic x-rays (weight-bearing) were taken. X-rays were evaluated and if no contraindications to thoracic manipulation were present the participant was scheduled for a baseline visit. At the baseline visit, a case history, EDSS assessment, chiropractic thoracic musculoskeletal examination, and blood sample were collected. Patient-reported outcome measures (pain, fatigue, depression, anxiety, subjective sleep) cognitive processing speed, and upper/lower limbs motor function were also assessed during the baseline visit. A tentative schedule of 8 visits over 4 weeks was arranged.

### 2.5 Blood sample protocol

**Figure 1:**
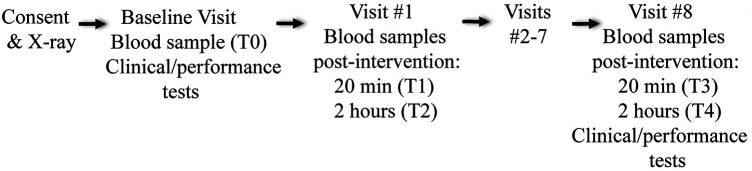
Study flow diagram.

Blood samples were collected approximately 1 week pre-intervention (T0, baseline), visit 1 (20 min [T1] and 2 h [T2] after intervention delivery), and visit 8 (20 min [T3] and 2 h [T4] after intervention delivery) (Figure 1). Visits 2–7 involved chiropractic thoracic SM group (including trigger point therapy) or sham-SM only. For blood sample collection, a phlebotomist from the UAB Clinical Research Unit collected 5 mL blood samples from the antecubital fossa vein or other anatomical site if necessary or preferred. Blood samples were drawn in commercially available 10 mL tubes using disposable catheters. Due to the length of time between blood sample collections and the non-restricted activity between sample collections, multiple needle sticks were used. Blood samples were left at room temperature for 45 min and then centrifuged at 3,400 RPM for 15 min at 4 °C in a refrigerated Eppendorf Centrifuge 5702 R (Eppendorf AG, Hamburg, Germany). Serum samples were collected, aliquoted, and stored at −80 °C for later analysis. In an attempt to control for diurnal variation in cytokine levels, blood samples were collected at approximately the same time of day (morning or afternoon) per participant across all visits, with individualized scheduling, as recommended by previous studies highlighting the impact of circadian rhythms on cytokine production (46, 47).

### 2.6 Biomarkers analysis

For cytokine analysis and quantification, 150 µL of each serum sample were sent to Eve Technologies (Eve Technologies Corp Calgary, AB, Canada) for analysis using Human High Sensitivity Expanded Panel T-Cell 21-Plex Discovery Assay. The 21-plex consisted of Fractalkine, granulocyte-macrophage colony-stimulating factor (GM-CSF), IFN-*γ*, IL-1β, IL-2, IL-4, interleukin-5 (IL-5), IL-6, interleukin-7 (IL-7), interleukin-8 (IL-8), IL-10, interleukin-12p70 (IL-12p70), interleukin-13 (IL-13), IL-17A, IL-21, interleukin-23 (IL-23), interferon-inducible T-cell alpha chemoattractant (ITAC), macrophage inflammatory protein-1 alpha (MIP-1*α*), macrophage inflammatory protein-1 beta (MIP-1β), macrophage inflammatory protein-3 alpha (MIP-3*α*), and TNF-α. This panel includes pro- and anti-inflammatory cytokines, cytokines with dual roles, chemokines, and growth factors. All serum samples were analyzed in duplicate. In addition to the cytokine/chemokine analysis, 150 µL separate serum samples were sent to Eve Technologies to analyze levels of neuroaxonal damage biomarkers GFAP, t-tau and NfL. Cytokines/chemokines were analyzed for all five timepoints (T0,T1,T2,T3,T4) and neuroaxonal damage biomarkers were analyzed only at timepoints T0 and T4.

### 2.7 Interventions

Participants in the chiropractic thoracic SM intervention group received thoracic spine palpation, soft tissue relaxation (trigger point therapy) and a manually delivered posterior-to-anterior high-velocity, low-amplitude (HVLA) spinal manipulation restricted to the thoracic spine while in a prone position on a Zenith 220 chiropractic adjusting table. Thoracic manipulation was performed by a licensed chiropractor with over 14 years clinical practice experience using the diversified (crossed bilateral hypothenar transverse process contact) chiropractic manipulation technique at clinically identified dysfunctional segments of the thoracic spine identified via x-ray and static/motion palpation. The presence or absence of joint cavitation during HVLA thrust was noted, and if a cavitation did not occur, then a second thoracic manipulative thrust was delivered. A maximum of two manipulative thrusts were delivered at any one segmental level and more than one thoracic vertebra was manipulated if clinically indicated. Mechanical forces applied during the thoracic HVLA spinal manipulation were not quantified.

Participants in the sham-SM group received thoracic spine palpation without soft tissue relaxation techniques and a simulated sham thoracic spinal manipulation designed to mimic the physical contact and clinician interaction of chiropractic treatment without delivering a therapeutic thrust. For the sham-SM, an Activator II device (Activator Methods®, Phoenix AZ) was used and set to zero (no force output) and placed onto the dorsal surface of the clinician's thumb, without direct instrument contact with the participant. A sham-SM was delivered at multiple non-vertebral trunk anatomical sites. The Activator II device is a spring-activated device that produces an audible clicking sound upon application but applies no force at a zero setting. This method of sham-SM has been used in previous studies with slight variation (48, 49), and serves as a control of participant-clinician physical contact, verbal interaction, and contextual factors during the office visit.

### 2.8 Outcomes measures

Outcome measures consisted of change scores in serum levels of 21 cytokines/chemokines (IL-1β, IL-2, IL-4, IL-5, IL-6, IL-7, IL-8, IL-10, IL-12p70, IL-13, IL-17A, IL-21, IL-23, Fractalkine, GM-CSF, IFN*γ*, ITAC, MIP-1*α*, MIP-1β, MIP-3*α*, TNF-α) and neuroaxonal biomarkers of GFAP, NfL, and t-tau from baseline. Other outcomes included patient-reported measures (pain, fatigue, depression, anxiety, subjective sleep), cognitive processing speed, and upper/lower limbs motor function. Pain was evaluated using the Short-Form McGill Pain Questionnaire (SF-MPQ), a 15-item instrument that measures sensory and affective dimensions of pain on a 0–3 scale, yielding total scores from 0 to 45 (higher scores indicating greater pain symptoms). The SF-MPQ also includes a Visual Analog Scale (VAS) ranging from 0 to 10, with higher scores indicating greater pain intensity (50). Fatigue was assessed through the Fatigue Severity Scale (FSS), a 9-item scale scored from 1 to 7 (higher scores indicating greater severity). The FSS also includes a Visual Analog Scale (VAS) ranging from 0 to 10, where higher scores reflect less fatigue (51). Additionally, the Modified Fatigue Impact Scale (MFIS), a 21-item measure, was used to assess fatigue across physical (0–36), cognitive (0–40), and psychosocial (0–8) domains, with a total score range of 0–84 (higher scores indicating greater impact of fatigue) (52). Mood symptoms were measured using the Hospital Anxiety and Depression Scale (HADS), a 14-item instrument with separate subscales for anxiety and depression (each scored 0–21) with higher scores indicating the probability of anxiety or depression (53). Sleep was evaluated using the Insomnia Severity Index (ISI), a 7-item scale with scores ranging from 0 to 28 (higher scores indicate greater insomnia) (54). Cognitive processing speed was measured using the Symbol Digit Modalities Test (SDMT), which requires participants to match symbols with corresponding numbers, with performance scored by the number of correct responses in 90 s (55). Motor function was assessed using the Nine-Hole Peg Test (NHPT) for upper limb dexterity, measuring the time to place and remove pegs averaged across two trials per hand (56), and the Timed 25-Foot Walk Test (T25FW) for walking speed, calculated as the average time to walk 25 feet over two trials. These outcomes were assessed at baseline and final visit (visit 8) and were performed to better inform which should be included in future SM RRMS clinical studies.

### 2.9 Data analysis

Participant demographic and baseline clinical characteristics were summarized using descriptive statistics. Continuous variables were reported as mean ± standard deviation (SD) and compared between two arms using independent samples t-test, while categorical variables were presented as median [IQR] and/or frequencies & percentages and compared using Fisher's Exact test and/or Independent-Samples Mann–Whitney U test. To evaluate treatment-related changes over time, individual change scores were calculated by subtracting baseline (T0) values from each subsequent timepoint. For cytokines/chemokines, which were measured at five timepoints, four change scores were calculated as *Δ*T1 = T1 – T0, *Δ*T2 = T2 – T0, *Δ*T3 = T3 – T0, *Δ*T4 = T4 – T0. For neurodegenerative biomarkers (GFAP, NfL, and t-tau), measured only at T0 and T4, change scores were calculated as *Δ* = T4 – T0. Similarly, clinical- and performance-based outcomes, which were assessed at baseline and final visit, were analyzed using change scores from baseline to the final visit. Between-group differences in mean change scores were calculated by subtracting the mean change in the treatment group from that of the sham-SM group. Given the exploratory nature of this pilot trial and the goal being to inform future work, no formal hypothesis testing was conducted. Instead, the analysis focused on estimating the magnitude and direction of group differences over time using effect sizes, calculated as Cohen's *d*, and 95% confidence intervals (CI). Effect sizes were interpreted according to standard thresholds (small ≥ 0.2, medium ≥ 0.5, large ≥ 0.8) (57). A positive effect size indicated an increase from baseline while a negative effect size indicated a decrease from baseline. All analyses were conducted using SPSS version 29.0 (IBM Corp, Armonk, NY).

## 3 Results

### 3.1 Participants

**Figure 2:**
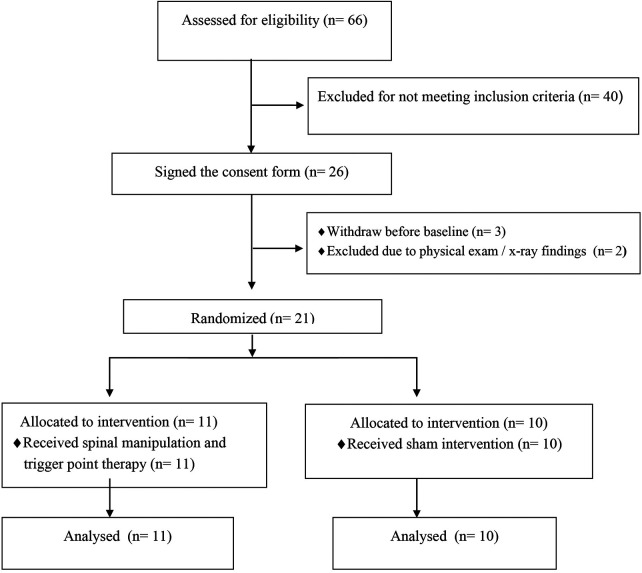
CONSORT flow diagram.

Recruitment began in September 2023 and data collection ended in February 2025. Sixty-six people with MS were screened for eligibility. Of these, 26 met the inclusion criteria and provided informed consent. Three participants withdrew before the baseline visit, and 2 were excluded due to physical exam/x-ray findings. All of 21 participants who were included completed the study: 11 in the SM group and 10 in the sham-SM group (Figure 2). There were no significant differences between the groups at baseline on demographic and clinical variables (Table 1).

**Table 1:** Baseline demographics and clinical characteristics:.

Characteristic	Total (*n* = 21)	Treatment (*n* = 11)	Sham (*n* = 10)	*P*-value
Age	42.4 ± 7.8	43.7 ± 8.9	41.1 ± 6.7	0.461^a^
Years since diagnosis	11.5 ± 6.2	11.9 ± 6.2	11.2 ± 6.5	0.803^a^
EDSS	2.0 [1.0,2.8]	1.5 [1.0,2.0]	2.0 [1.0,3.1]	0.426^b^
Sex, *n* (%)				>0.999^c^
Female	15 (71.4%)	8 (72.7%)	7 (70.0%)	
Male	6 (28.6%)	3 (27.3%)	3 (30.0%)	
Race, *n* (%)				0.183^c^
White	8 (38.1%)	6 (54.5%)	2 (20.0%)	
Black	12 (57.1%)	5 (45.5%)	7 (70.0%)	
Unknown	1 (4.8%)	0 (0.0%)	1 (10.0%)	
Ethnicity, *n* (%)				0.361^c^
Hispanic	1 (4.8%)	0 (0.0%)	1 (10.0%)	
Non-Hispanic	7 (33.3%)	5 (45.5%)	2 (20.0%)	
Unknown	13 (61.9%)	6 (54.5%)	7 (70.0%)	

Data are presented as mean ± SD, median [IQR], number (percentage).

^a^ independent sample t test,

^b^ Independent-Samples Mann–Whitney U test,

^c^ Fisher's Exact test.

EDSS: Expanded Disability Status Scale.

### 3.2 Inflammatory biomarkers

Mean serum levels of inflammatory biomarkers at baseline and each post-intervention timepoint are provided in Supplement Table S1. The mean change scores from baseline for each timepoint, between-group mean difference [95% CI], and effect sizes are provided in Table 2. A summary of inflammatory biomarkers effect sizes by post-intervention timepoints are provided in Table 3.

**Table 2:** Mean change in serum cytokines levels from baseline by group, with between-group difference and effect size.

Biomarker	Change From Baseline	Treatment (*n* = 11)	Sham (*n* = 10)	Mean Difference [95%CI]	Cohen's d	Biomarker	Change From Baseline	Treatment (*n* = 11)	Sham (*n* = 10)	Mean Difference [95%CI]	Cohen's d
IL-1β	T1 – T0	0.13 (0.35)	0.07 (0.23)	0.06 [−0.21, 0.33]	0.20	IL-21	T1 – T0	-0.07 (0.47)	0.11 (1.00)	-0.19 [−0.89, 0.52]	-0.24
	T2 – T0	0.03 (0.36)	0.05 (0.13)	-0.02 [−0.27, 0.23]	-0.07		T2 – T0	0.43 (1.00)	-0.05 (0.51)	0.48 [−0.25, 1.21]	0.60
	T3 – T0	0.06 (0.35)	0.06 (0.30)	-0.00 [−0.31, 0.30]	-0.01		T3 – T0	-0.06 (0.84)	0.29 (0.91)	-0.35 [−1.15, 0.45]	-0.40
	T4 – T0	0.06 (0.26)	0.02 (0.46)	0.04 [−0.30, 0.38]	0.10		T4 – T0	0.72 (1.40)	0.15 (0.88)	0.57 [−0.51, 1.65]	0.48
IL-2	T1 – T0	-0.09 (0.64)	0.01 (0.18)	-0.10 [−0.54, 0.34]	-0.21	IL-23	T1 – T0	11.45 (63.04)	5.89 (23.24)	5.56 [−38.74, 49.87]	0.12
	T2 – T0	0.25 (0.61)	0.23 (0.53)	0.02 [−0.51, 0.54]	0.03		T2 – T0	16.15 (57.60)	0.33 (42.61)	15.83 [−30.86, 62.51]	0.31
	T3 – T0	0.05 (0.34)	0.30 (1.38)	-0.25 [−1.15, 0.64]	-0.26		T3 – T0	-19.53 (102.22)	8.29 (44.92)	-27.82 [−101.30, 45.65]	-0.35
	T4 – T0	0.13 (0.43)	0.01 (1.12)	0.12 [−0.64, 0.88]	0.14		T4 – T0	12.56 (66.59)	-10.18 (49.44)	22.74 [−31.30, 76.78]	0.39
IL-4	T1 – T0	1.86 (8.25)	2.54 (9.12)	-0.68 [−8.61, 7.25]	-0.08	Fractalkine	T1 – T0	-0.02 (14.65)	0.82 (8.65)	-0.84 [−11.98, 10.30]	-0.07
	T2 – T0	-0.15 (11.25)	5.06 (14.71)	-5.21 [−17.10, 6.67]	-0.40		T2 – T0	1.87 (15.61)	1.67 (5.81)	0.20 [−10.78, 11.18]	0.02
	T3 – T0	-5.00 (11.06)	-0.40 (2.69)	-4.60 [−12.15, 2.95]	-0.56		T3 – T0	4.38 (9.70)	-1.82 (6.35)	6.20 [−1.38, 13.78]	0.75
	T4 – T0	1.99 (4.87)	2.02 (5.33)	-0.03 [−4.69, 4.62]	-0.01		T4 – T0	4.85 (11.14)	-2.64 (15.64)	7.50 [−4.81, 19.80]	0.56
IL-5	T1 – T0	0.08 (0.21)	0.06 (0.23)	0.03 [−0.17, 0.23]	0.12	GM-CSF	T1 – T0	1.39 (3.37)	-0.45 (1.60)	1.84 [−0.62, 4.29]	0.68
	T2 – T0	0.19 (0.36)	0.07 (0.23)	0.11 [−0.16, 0.39]	0.38		T2 – T0	2.41 (3.30)	-0.62 (1.49)	3.03 [0.65, 5.41]	1.17
	T3 – T0	0.01 (0.25)	-0.04 (0.13)	0.05 [−0.13, 0.23]	0.24		T3 – T0	1.69 (4.24)	-1.06 (1.28)	2.75 [−0.17, 5.67]	0.86
	T4 – T0	0.14 (0.31)	-0.04 (0.30)	0.18 [−0.10, 0.46]	0.60		T4 – T0	2.51 (4.20)	-1.31 (3.46)	3.82 [0.28, 7.36]	0.99
IL-6	T1 – T0	0.11 (0.42)	0.11 (0.25)	0.01 [−0.32, 0.33]	0.02	IFN*γ*	T1 – T0	0.23 (0.96)	0.11 (0.68)	0.12 [−0.65, 0.89]	0.14
	T2 – T0	0.02 (0.52)	0.27 (0.47)	-0.24 [−0.70, 0.22]	-0.48		T2 – T0	0.50 (1.02)	0.19 (0.65)	0.31 [−0.48, 1.10]	0.36
	T3 – T0	-0.36 (1.34)	0.16 (0.52)	-0.52 [−1.47, 0.43]	-0.50		T3 – T0	0.41 (1.21)	-0.09 (0.59)	0.50 [−0.38, 1.39]	0.52
	T4 – T0	-0.04 (0.58)	0.19 (0.61)	-0.24 [−0.78, 0.30]	-0.40		T4 – T0	0.91 (2.01)	-0.06 (1.64)	0.97 [−0.72, 2.66]	0.53
IL-7	T1 – T0	0.00 (0.88)	-0.05 (0.52)	0.06 [−0.61, 0.73]	0.08	ITAC	T1 – T0	-0.85 (10.05)	-1.50 (4.96)	0.66 [−6.70, 8.02]	0.08
	T2 – T0	0.20 (1.03)	-0.13 (0.51)	0.33 [−0.42, 1.08]	0.40		T2 – T0	0.15 (7.37)	-0.32 (4.81)	0.47 [−5.28, 6.22]	0.07
	T3 – T0	0.30 (1.29)	-0.44 (0.84)	0.74 [−0.27, 1.74]	0.67		T3 – T0	3.55 (25.32)	-2.10 (6.59)	5.64 [−11.66, 22.95]	0.30
	T4 – T0	0.17 (1.12)	-0.20 (0.85)	0.37 [−0.54, 1.29]	0.38		T4 – T0	4.16 (19.60)	-2.89 (5.48)	7.05 [−6.41, 20.50]	0.48
IL-8	T1 – T0	0.97 (1.69)	-1.21 (3.02)	2.18 [−0.03, 4.39]	0.90	MIP-1*α*	T1 – T0	9.15 (28.14)	0.06 (1.22)	9.09 [−9.59, 27.78]	0.45
	T2 – T0	0.47 (1.69)	-1.09 (2.93)	1.56 [−0.60, 3.73]	0.66		T2 – T0	-3.84 (16.59)	-0.58 (2.41)	-3.26 [−14.37, 7.86]	-0.27
	T3 – T0	-0.82 (3.03)	-1.34 (2.97)	0.52 [−2.23, 3.26]	0.17		T3 – T0	5.70 (17.99)	-0.54 (2.01)	6.23 [−5.77, 18.23]	0.47
	T4 – T0	-0.12 (2.00)	-0.52 (1.67)	0.40 [−1.29, 2.10]	0.22		T4 – T0	5.98 (11.77)	-1.58 (3.77)	7.56 [−0.60, 15.72]	0.85
IL-10	T1 – T0	0.14 (1.26)	-0.05 (0.60)	0.18 [−0.74, 1.10]	0.18	MIP-1β	T1 – T0	1.03 (4.97)	-0.28 (3.05)	1.31 [−2.51, 5.12]	0.31
	T2 – T0	0.30 (0.47)	-0.02 (0.78)	0.32 [−0.26, 0.90]	0.51		T2 – T0	3.10 (6.12)	-0.42 (2.78)	3.52 [−0.86, 7.90]	0.73
	T3 – T0	-0.95 (2.59)	-0.10 (0.71)	-0.85 [−2.62, 0.93]	-0.43		T3 – T0	-1.18 (8.07)	-1.31 (3.02)	0.14 [−5.54, 5.82]	0.02
	T4 – T0	0.51 (2.16)	0.24 (1.09)	0.27 [−1.32, 1.85]	0.15		T4 – T0	1.88 (5.75)	-2.87 (4.01)	4.75 [0.18, 9.33]	0.95
IL-12p70	T1 – T0	-0.24 (0.79)	-0.23 (0.74)	-0.02 [−0.72, 0.69]	-0.02	MIP-3α	T1 – T0	20.74 (69.04)	1.00 (2.29)	19.75 [−26.08, 65.57]	0.39
	T2 – T0	0.02 (0.50)	-0.14 (0.74)	0.16 [−0.40, 0.73]	0.27		T2 – T0	9.33 (24.87)	0.93 (3.58)	8.40 [−8.25, 25.05]	0.46
	T3 – T0	-0.20 (0.64)	-0.32 (0.26)	0.12 [−0.33, 0.58]	0.25		T3 – T0	0.53 (4.66)	1.02 (2.86)	-0.49 [−4.07, 3.09]	-0.12
	T4 – T0	-0.03 (0.47)	-0.22 (0.53)	0.19 [−0.27, 0.65]	0.38		T4 – T0	1.25 (1.86)	0.05 (3.97)	1.20 [−1.59, 3.99]	0.39
IL-13	T1 – T0	0.23 (0.91)	-0.70 (1.68)	0.93 [−0.28, 2.15]	0.70	TNF-α	T1 – T0	-0.02 (0.99)	-0.18 (0.71)	0.16 [−0.63, 0.95]	0.18
	T2 – T0	-0.27 (2.32)	-0.25 (1.03)	-0.02 [−1.69, 1.65]	-0.01		T2 – T0	0.31 (0.96)	-0.02 (0.65)	0.33 [−0.43, 1.09]	0.40
	T3 – T0	-1.14 (2.87)	-0.74 (2.73)	-0.40 [−2.96, 2.17]	-0.14		T3 – T0	0.08 (1.20)	0.01 (0.74)	0.07 [−0.84, 0.98]	0.07
	T4 – T0	-0.36 (1.49)	-0.50 (2.53)	0.15 [−1.73, 2.02]	0.07		T4 – T0	0.17 (1.10)	-0.31 (1.01)	0.49 [−0.49, 1.46]	0.46
IL-17A	T1 – T0	0.76 (1.68)	-0.04 (1.73)	0.79 [−0.77, 2.35]	0.47						
	T2 – T0	1.16 (2.14)	-0.32 (1.48)	1.47 [−0.23, 3.17]	0.79						
	T3 – T0	1.58 (3.37)	-0.77 (1.56)	2.35 [−0.09, 4.80]	0.88						
	T4 – T0	1.75 (2.66)	-0.96 (3.20)	2.70 [0.02, 5.38]	0.92						

Cytokine concentrations are reported in pg/mL. Data are presented as mean (SD). T0 = baseline; T1 = 20 min after first intervention; T2 = 2 h after first intervention; T3 = 20 min after the final intervention; T4 = 2 h after the final intervention. IL, interleukin; GM-CSF, granulocyte-macrophage colony-stimulating factor; IFNγ, interferon gamma; ITAC, interferon-inducible T-cell alpha chemoattractant; MIP-1α, macrophage inflammatory protein-1 alpha; MIP-1β, macrophage inflammatory protein-1 beta; MIP-3α, macrophage inflammatory protein-3 alpha; TNF-α, tumor necrosis factor alpha.

**Table 3:** Inflammatory biomarkers effect sizes by post-intervention time points. .

Effect size	T1	T2	T3	T4
Large (d ≥ 0.8)	IL-8	GM-CSF	IL-17A GM-CSF	IL-17A MIP-1α MIP-1β GM-CSF
Moderate (d ≥ 0.5)	IL-13 GM-CSF	IL-8 IL-10 IL-21 IL-17A MIP-1β	IL-4 IL-6 IL-7 IFNγ Fractalkine	IL-5 IFNγ Fractalkine
Small (d ≥ 0.2)	IL-1β IL-2 IL-21 IFNγ ITAC MIP-1α MIP-1β MIP-3α TNF-α IL-17A	IL-4 IL-5 IL-6 IL-7 IL-12p70 IL-23 IFNγ MIP-1α MIP-3α TNF-α	IL-2 IL-5 IL-10 IL-12p70 IL-21 IL-23 ITAC MIP-1α	IL-6 IL-7 IL-8 IL-12p70 IL-21 IL-23 ITAC MIP-3α TNF-α
Very Small (d < 0.2)	IL-4 IL-5 IL-6 IL-7 IL-10 IL-12p70 IL-23 Fractalkine IFNγ ITAC	IL-1β IL-2 IL-13 Fractalkine ITAC	IL-1β IL-8 IL-13 MIP-1β MIP-3α TNF-α	IL-1β IL-2 IL-4 IL-10 IL-13

T0 = baseline; T1 = 20 min after first intervention; T2 = 2 h after first intervention; T3 = 20 min after the final intervention; T4 = 2 h after the final intervention.. IL, interleukin; GM-CSF, granulocyte-macrophage colony-stimulating factor; IFNγ, interferon gamma; ITAC, interferon-inducible T-cell alpha chemoattractant; MIP-1α, macrophage inflammatory protein-1 alpha; MIP-1β, macrophage inflammatory protein-1 beta; MIP-3α, macrophage inflammatory protein-3 alpha; TNF-α, tumor necrosis factor alpha.

For the majority of cytokines/chemokines analyzed, between-group effect size changes of inflammatory biomarkers were small to negligible (Table 2). Eight cytokines/chemokines demonstrated moderate to large effect size changes at a single post-intervention timepoint (IL-13 [T1], IL-10 and IL-21 [T2], IL-4, IL-6, IL-7 [T3], and IL-5 and MIP-1*α* [T4] (Table 3). Whereas 6 of 21 cytokines/chemokines demonstrated between-group moderate to large effect sizes at multiple timepoints (IL-8, IL-17A GM-CSF, MIP-1β, IFN*γ*, Fractalkine) (Table 3). GM-CSF was the only biomarker to have a moderate to large effect size at all four post-intervention timepoints assessed, as well as having the largest effect size (d = 1.17). IL-17A demonstrated moderate to large effect sizes at three of the four timepoints assessed and was the only biomarker that demonstrated a progressive increase of between-group differences across all timepoints (d = 0.47 [T1], 0.79 [T2], 0.88 [T3], and 0.92 [T4]) (Table 2). IFN*γ* displayed moderate effect size T3 and T4, while MIP-1β demonstrated a moderate effect size at T2 and large effect size at T4 (Table 3). Among anti-inflammatory cytokines, IL-13 showed a moderate effect size at T1, and IL-10 at T2, while IL-4 demonstrated a negative moderate effect size at T3. IL-6 is the only other cytokine to demonstrate a moderate to large negative effect size, and similar to IL-4 this decrease occurred at T3 (Table 3). Despite IL-6 reaching a negative moderate effect size at only one timepoint, timepoints T2 (d = −0.48) and T4 (d = −0.40) reached a near moderate effect size. Another dual function cytokine, IL-21 reached a moderate effect at T2 (Table 3). IL-2, IL-1β, IL-12p70, IL-23, ITAC, MIP-3*α*, and TNF-α all showed small or negligible between-group differences and small effect sizes at all timepoints evaluated (Table 2).

### 3.3 Neuroaxonal damage biomarkers

Mean serum levels of neuroaxonal damage biomarkers at each measurement time, the mean change from baseline, between-group mean difference [95% CI], and effect sizes are provided in Table 4. Small effect sizes were observed for GFAP (*d* = 0.29) and NfL (*d* = 0.21), while t-tau demonstrated a nearly moderate negative effect (*d* = −0.42) indicating a decrease in tau levels in the SM group.

**Table 4:** Group means, change scores from baseline, and between-group differences with effect sizes for neurodegenerative biomarkers.

Biomarker	Time point	Treatment (*n* = 11)	Change Score (T4-T0)	Sham (*n* = 10)	Change Score (T4-T0)	Mean Difference [95%CI]	Cohen's d
GFAP	T0	48164 (29537)	2845 (7066)	26146 (10377)	1127 (4078)	1718 [−3627, 7063]	0.29
	T4	51008 (30938)		27272 (12063)			
NFL	T0	63986 (60066)	7207 (17498)	32639 (7043)	4277 (6570)	2930 [−9394, 15254]	0.21
	T4	71193 (74056)		36916 (7414)			
Tau	T0	10754 (2972)	−209 (2914)	10803 (3908)	1534 (5082)	−1743 [−5481, 1994]	−0.42
	T4	10545 (3377)		12337 (5724)			

Neurodegenerative biomarker concentrations are reported in fg/mL. Data are presented as mean (SD). GFAP, Glial Fibrillary Acidic Protein; NfL, Neurofilament Light; Tau, Total-tau. T0 = baseline; T4 = 2 h after the final intervention.

### 3.4 Clinical/performance-based outcomes

Mean change from baseline, between-group mean difference [95% CI] and effect sizes for patient reported clinical/performance-based outcomes are provided in Table 5. Of the clinical/performance outcomes evaluated, 66.7% (8/12) demonstrated negligible changes yielding small effect sizes, while the remaining four outcomes had moderate effects. None of the clinical-and performance-based outcomes demonstrated large effect sizes.

**Table 5:** Group means, change scores from baseline, and between-group differences with effect sizes for clinical/performance-based outcomes.

Outcome measure	Time	Treatment (*n* = 11)	Change Score	Sham (*n* = 10)	Change Score	Mean Difference [95%CI]	Cohen's d
HADS-Depression	Baseline	4.91 (4.74)	−0.09 (1.51)	8.40 (3.37)	−1.20 (2.25)	1.11 [−0.63, 2.85]	0.58
	Visit 8	4.82 (4.77)		7.20 (2.66)			
HADS-Anxiety	Baseline	6.36 (4.18)	−0.18 (2.18)	9.20 (3.74)	−0.50 (2.92)	0.32 [−2.02, 2.66]	0.12
	Visit 8	6.18 (4.69)		8.70 (4.81)			
ISI	Baseline	9.82 (7.21)	1.55 (5.77)	11.90 (5.13)	−0.70 (5.79)	2.25 [−3.04, 7.53]	0.38
	Visit 8	11.36 (7.24)		11.20 (6.58)			
SF-MPQ	Baseline	0.55 (1.81)	0.91 (4.21)	8.70 (10.69)	−5.50 (11.94)	6.41 [−1.61, 14.42]	0.73
	Visit 8	1.45 (3.56)		3.20 (3.29)			
Pain VAS	Baseline	0.36 (1.21)	0.91 (3.42)	4.90 (2.81)	−1.50 (3.81)	2.41 [−0.89, 5.71]	0.66
	Visit 8	1.27 (3.04)		3.40 (2.95)			
FSS	Baseline	3.98 (1.09)	0.21 (0.76)	4.36 (1.46)	−0.20 (1.06)	0.41 [−0.43, 1.25]	0.44
	Visit 8	4.19 (1.42)		4.16 (1.04)			
Fatigue VAS	Baseline	5.91 (3.05)	−0.73 (3.13)	5.10 (2.81)	−0.30 (2.00)	−0.43 [−2.86, 2.00]	−0.16
	Visit 8	5.18 (3.49)		4.80 (2.25)			
MFIS	Baseline	32.27 (18.93)	1.00 (12.98)	46.30 (11.57)	−0.90 (10.85)	1.90 [−9.09, 12.89]	0.15
	Visit 8	33.27 (23.19)		45.40 (12.62)			
SDMT	Baseline	54.09 (18.15)	2.91 (6.66)	51.10 (8.69)	2.90 (8.24)	0.01 [−6.80, 6.82]	0.001
	Visit 8	57.00 (20.21)		54.00 (7.62)			
NHPT-DH	Baseline	20.66 (4.79)	0.65 (2.64)	22.18 (4.47)	−0.71 (2.28)	1.37 [−0.90, 3.63]	0.55
	Visit 8	21.31 (6.63)		21.47 (3.64)			
NHPT-NDH	Baseline	23.09 (6.07)	−0.38 (2.69)	22.68 (3.12)	−0.32 (2.58)	−0.06 [−2.48, 2.35]	−0.02
	Visit 8	22.71 (6.37)		22.36 (2.88)			
T25FW Test	Baseline	5.27 (1.96)	−0.28 (0.51)	5.69 (1.18)	−0.35 (0.46)	0.07 [−0.38, 0.51]	0.13
	Visit 8	4.99 (1.74)		5.35 (1.11)			

Data are presented as mean (SD). HADS, Hospital Anxiety and Depression Scale; ISI, Insomnia Severity Index; SF-MPQ, Short-form McGill Pain Questionnaire; VAS, Visual Analog Scale; FSS, Fatigue Severity Scale; MFIS, Modified Fatigue Impact Scale; SDMT, Symbol Digit Modalities Test; NHPT-DH, Nine-hole Peg Test for dominant hand; NHPT-NDH, Nine-hole Peg Test for Non Dominant Hand; T25FW, Timed 25-foot Walk Test.

Fatigue measures (FSS, MFIS, and fatigue VAS) demonstrated small effect sizes, with slight improvements favoring the sham group and no meaningful between-group differences. Anxiety (HADS) and insomnia (ISI) scores also showed small changes in both groups, with minimal between-group differences and small effect sizes. Cognitive performance (SDMT) improved equally in both groups with a negligible effect size (*d* = 0.001). Of the four clinical- and performance-based outcomes with moderate effect sizes, depression (HADS) scores slightly improved in both groups, with a small between-group difference and moderate effect size favoring the sham group (*d* = 0.58). Pain symptoms (SF-MPQ) and pain intensity (Pain VAS) both increased in the treatment group while decreasing in the sham group, with moderate effects (*d* = 0.73 and 0.66, respectively). Motor function outcomes showed a modest benefit in the sham group for the dominant hand (NHPT) (*d* = 0.55); however non-dominant hand use (NHPT) and walking speed (T25FW) showed negligible group differences and a small effect sizes (*d* = −0.02 and 0.13, respectively).

## 4 Discussion

To our knowledge, this is the first investigation of the effects of chiropractic thoracic SM on cytokine/chemokine biomarkers in MS, a chronic neurological disease characterized by inflammation, demyelination, and neuroaxonal loss within the CNS. This pilot study evaluated the intermediate (20 min) and short-term (2 h) effects of a single, as well as a short series of thoracic SMs including trigger point therapy on serum cytokine/chemokine, neurodegenerative biomarkers and clinical/performance outcomes in people with RRMS. The goal of this pilot study was to identify cytokines/chemokines, neurodegenerative biomarkers and clinical- and performance-based tests that should be investigated further in appropriately powered RRMS clinical trials involving SM/trigger point therapy. In the current pilot study, the majority of the 21 inflammatory biomarkers assessed had small or negligible between-group effect size at post-intervention timepoints (Table 3). However, a subset of inflammatory cytokines/chemokines demonstrated moderate-to-large effect size changes at either one post-intervention timepoint (IL-4, IL-5, IL-6, IL-7, IL-13, IL-10, IL-21, MIP-1*α*) or at multiple timepoints (IL-8, IL-17A, IFN*γ*, GM-CSF, MIP-1β, Fractalkine) suggesting potential responses related to SM and/or the accompanying trigger point therapy. Among the eight cytokines/chemokines having a moderate effect size at a single post-intervention timepoint, three are considered anti-inflammatory (IL-4, IL-10, IL-13), however IL-4 is one of two cytokines that demonstrated negative changes. IL-6 was the only other cytokine/chemokine demonstrating a negative change of moderate effect size with SM. While IL-6 is considered to be primarily pro-inflammatory, it does exhibit anti-inflammatory properties. Both IL-6 and TNF-α have been implicated in the breakdown of the blood brain barrier (58), thus small (TNF-α) and negative (IL-6) changes as seen in this pilot should be considered potentially positive. Pro-inflammatory cytokines/chemokines demonstrating a moderate to large effect size at a single timepoint included: IL-5, IL-7, IL-21, and MIP-1*α*. These pro-inflammatory cytokines/chemokines were distributed across all post-intervention timepoints without a pattern emerging. Cytokines/chemokines demonstrating moderate to large effect sizes at a single post-intervention timepoint must be interpreted with caution due to the established variability and natural fluctuations known to exist among cytokines/chemokines and moderate-to-larger effect sizes represent exploratory signals rather than evidence of clinical benefit or detriment.

A closer examination of the 6 cytokine/chemokines (IL-8, IL-17A, IFN*γ*, GM-CSF, MIP-1β, Fractalkine) demonstrating changes of moderate to large effect size at multiple post-intervention timepoints of the SM group is warranted. Among the most consistent post-intervention findings were changes in GM-CSF (granulocyte-macrophage colony-stimulating factor), which had the largest overall effect size (d = 1.17), and IL-17A. GM-CSF is the only biomarker that demonstrated moderate-to-large between-group effect sizes at all post-intervention timepoints, whereas IL-17A had a progressive increase in between-group differences across three of the four timepoints (Table 3). It is interesting to note that GM-CSF expression is regulated by IL-23 and IL-1β (59, 60), both of which had negligible effect size changes with SM. Both GM-CSF and IL-17A are functionally linked to T helper 17 (Th17) lineage and are strongly implicated in the pathogenesis of MS (61–64). In contrast, other Th17-related cytokines such as IL-21 exhibited only a moderate effect size at a single timepoint (Table 3). These findings suggest a degree of functional heterogeneity within the Th17 lineage response to SM. GM-CSF originates from haematopoietic cells, epithelial cells, fibroblasts and stromal cells and is rarely detected in healthy physiological conditions (59). Since fibroblasts are a primary cell type of myofascia, one cannot help to speculate whether the myofascial trigger point work performed prior to nearly all SM treatments acted to increase GM-CSF levels in the SM group. Using a microdialysis technique in the trapezius and gastrocnemius muscles, individuals with myofascial trigger points were shown to have higher concentrations of TNF-α, IL-1β, IL-6, IL-8 (among other inflammatory-related analytes) compared with those who did not (65). Therefore, future SM in RRMS studies may wish to restrict trigger point or other soft work prior to SM delivery. Among the Th1 cytokines (IL-1β, IL-2, IL-12p70, TNF-α, IFN*γ*), also known to be important drivers of inflammation in MS population (64, 66), only IFN*γ* (interferon gamma) showed a moderate to large effect size at any post-intervention timepoint. Fractalkine and MIP-1β are also both considered pro-inflammatory. Therefore, while the majority of the cytokines/chemokines exhibiting moderate to large effect size changes in relation to sham-SM are considered inflammatory, it must not be forgotten that many other highly recognized inflammatory mediators such as TNF-α, IL-6, IL-1β, IL-2, and IL-12p70 demonstrated negligible to small effect sizes, with IL-6 demonstrating a decrease in three of the four post-intervention timepoints assessed (Table 2). It should be noted that the current pilot study only assessed 21 cytokines/chemokines, while other recent MS studies assessed much larger numbers of serum cytokines/chemokines including 36 (67), 45 (12), 48 (68), 65 (13), and therefore a comprehensive assessment of inflammatory cytokine/chemokine will require additional investigation.

Of fifteen randomized controlled trials included in a recent systematic review evaluating biochemical changes following the single modality of SM, only four reported the effects of SM on inflammatory cytokines/chemokines (192 participants; not pooled) (34). The authors concluded that these four studies provided low quality evidence suggesting that SM as a single modality is better than control in influencing various inflammatory markers (especially IL-1, IL-2, IL-6, TNF-α). However, the authors urged caution based on the inclusion of healthy participants, small sample sizes, and/or low number of studies performed in the last decade (34). The authors recognized that the review excluded studies involving the more clinically relevant multi-modal therapeutic approaches that included SM among other types of non-pharmacological treatment. Licciardone et al. (69) investigated the effects of a multi-modal approach of osteopathic manipulative techniques (including SM, myofascial stretching and release, trigger point therapy, etc.) and reported a reduction in TNF-α compared to control, while finding no significant changes in IL-1β, IL-6, IL-8, IL-10. Other SM-related studies in patients with LBP also report reduction in TNF-α following manual therapy treatments that decreased symptoms (39, 40, 69, 70). These findings were contrary to that reported in other studies (42). A recent systematic review of changes in inflammatory biomarkers after non-pharmalogical interventions for chronic LBP evaluated 3 SM-related studies which taken together reported significant decreases in IL-6, C-reactive protein (CRP), MIP-1*α*, while no significant changes in IL-1β, IL-2, IL-10, IFN*γ*, TNF-α and monocyte chemoattractant protein-1 (MCP-1) (35). While current findings remain somewhat mixed in SM-related studies of individuals with LBP, the fact that more SM-related biomarker studies are being conducted in symptomatic populations is encouraging and will move the field forward.

Regarding neurodegeneration biomarkers, changes were minimal overall. Tau levels had a small to approaching moderate negative effect size (*d* = −0.42) indicating decreased t-tau levels in the SM group. GFAP and NfL, on the other hand, had small effect sizes. This is consistent with research indicating that these biomarkers undergo measurable changes in the context of long-term disease progression rather than following brief intervention (71).

Clinical- and performance-based outcomes in this pilot study demonstrated no clear benefit of a short course of thoracic SM/trigger point therapy. Although moderate effect sizes were observed for pain, depression, and dominant hand use, these differences favored the sham-SM group. Other motor outcomes, cognitive performance, as well as patient-reported measures of anxiety and fatigue did not demonstrate meaningful between-group differences and failed to meet their respective minimal detectable change thresholds in the MS population (72–76), suggesting that the observed changes would not likely be clinically relevant. Inclusion of low baseline EDSS scores and preserved ambulation likely limited the sensitivity of T25FW to detect changes over such a short intervention period. It was noted that physical symptoms such as pain and fatigue appeared to often fluctuate visit to visit depending on the amount of physical activity performed earlier that day or the day preceding the visit. Longer courses and/or whole spine SM treatment will likely be required to demonstrate improvement in clinical/performance-based outcomes in a RRMS population.

Future fully powered SM studies in RRMS should evaluate those cytokines/chemokines that demonstrated a moderate to large effect size as well as assessing more immediate biomarker changes (<5 min), in addition to the intermediate changes (20 min) and short duration (≥2 hrs) changes evaluated in the current pilot study. Since these cytokines were primarily inflammatory in nature, future studies should make a decision to restrict the intervention to just a single modality (SM) or to maintain a more clinically pragmatic multi-modal approach that includes the use of soft tissue relaxation techniques (i.e., trigger point therapy, muscle stretch, etc.) Multi-modal approaches may result in greater release of pro-inflammatory cytokines/chemokines compared to SM delivery alone. Future studies should plan to evaluate biomarker findings based on disease duration, disease severity, and specific pharmacological treatment.

## 5 Limitations

This pilot study has several limitations that should be considered when interpreting the findings. These limitations include: the exploratory nature and small sample size (*n* = 21) of this pilot study which was not adequately powered for hypothesis testing, but designed to inform for future studies; the timing of blood collection at 20 min and 2 h post-intervention may not have captured the immediate, earliest or most relevant changes in cytokine/chemokine levels related to SM. In addition, cytokines in this study were analyzed by Eve Technologies in two separate assays to minimize freezer storage time for samples; however, some interplate variation may have occurred. Furthermore, although all blood samples were collected at approximately the same time of day, participants could schedule their blood collection visits in either the morning or afternoon which may have introduced additional variability. Future studies should consider collecting all blood samples in a set window, (i.e., 8am - 10am), and perhaps request participants to restrict the amount of physical activity in the hours preceding the sample collection and detail the specific disease modification therapy (type and dosage). Chiropractic spinal manipulation in this study was restricted to the thoracic spine instead of full spine care as typically performed clinically. Future studies will need to investigate the cytokine/chemokine effects of full spine treatment. Inclusion of trigger point therapy prior to thoracic spinal manipulation, while clinically pragmatic, added complexity to the interpretation of the study findings as they could not be attributed exclusively to the spinal manipulation intervention. A relapse-free period of 3–6 months should be considered in future studies. Collecting greater detail of participant concomitant medications (i.e., antidepressants, anxiolytics, corticosteroid exposure, etc.) and disease-modifying therapies (type, duration, etc.) in future studies may be beneficial as well as these factors may impact biomarker and/or patient-reported outcomes.

## 6 Conclusion

A majority of the 21 cytokines/chemokines investigated in this pilot study demonstrated a negligible to small effect size with thoracic SM/trigger point therapy, with a subset of 6 pro-inflammatory cytokines/chemokines demonstrating changes at multiple timepoints post-intervention of moderate to large effect size. GM-CSF and IL-17A demonstrated the most consistent and largest effect size changes. All three neurodegenerative markers had small effect size changes with only Tau having a negative change approaching a moderate effect size. Clinical/performance-based measures demonstrated primarily small effect size or non-clinically meaningful changes. Additional study of the effects of SM will be needed to confirm and expand on these pilot study findings of neuroimmune changes in this RRMS population.

## Data Availability

The original contributions presented in the study are included in the article/Supplementary Material, further inquiries can be directed to the corresponding author.
