# Supplementary material for: Effects of thoracic manipulation with trigger point therapy on inflammatory cytokine levels in individuals with multiple sclerosis: a pilot study
Source: Front Rehabil Sci. 2026 Apr 8;7:1699274. doi: 10.3389/fresc.2026.1699274 (PMC13099762; doi:10.3389/fresc.2026.1699274)
Supplement: Supplementary file 1 [file Table1.docx]

**Supplementary Table 1. Mean serum cytokine levels at each time point.**

| **Bio-marker** | **Time Point** | **Treatment (n=11)** | **Sham (n=10)** |  | **Bio-**  **marker** | **Time Point** | **Treatment (n=11)** | **Sham (n=10)** |  | **Bio-marker** | **Time Point** | **Treatment (n=11)** | **Sham (n=10)** |
| --- | --- | --- | --- | --- | --- | --- | --- | --- | --- | --- | --- | --- | --- |
| IL-1β | T0 | 3.05 (3.64) | 2.12 (2.69) |  | IL-10 | T0 | 11.50 (23.47) | 8.58 (17.64) |  | GM-CSF | T0 | 23.95 (42.27) | 12.43 (12.79) |
|  | T1 | 3.18 (3.64) | 2.19 (2.83) |  |  | T1 | 11.64 (22.85) | 8.53 (17.55) |  |  | T1 | 25.34 (41.57) | 11.98 (12.04) |
|  | T2 | 3.09 (3.60) | 2.18 (2.78) |  |  | T2 | 11.80 (23.29) | 8.56 (17.68) |  |  | T2 | 26.35 (43.10) | 11.80 (12.53) |
|  | T3 | 3.11 (3.70) | 2.19 (2.98) |  |  | T3 | 10.56 (21.24) | 8.48 (17.36) |  |  | T3 | 25.63 (42.65) | 11.36 (12.74) |
|  | T4 | 3.11 (3.59) | 2.15 (3.00) |  |  | T4 | 12.01 (25.01) | 8.82 (17.14) |  |  | T4 | 26.45 (42.04) | 11.11 (13.43) |
| IL-2 | T0 | 4.02 (5.41) | 3.22 (4.65) |  | IL-12p70 | T0 | 3.54 (5.19) | 3.35 (4.78) |  | IFNγ | T0 | 8.22 (12.66) | 6.54 (9.75) |
|  | T1 | 3.93 (4.99) | 3.23 (4.59) |  |  | T1 | 3.30 (4.65) | 3.12 (4.34) |  |  | T1 | 8.45 (13.01) | 6.64 (10.01) |
|  | T2 | 4.27 (5.71) | 3.46 (4.97) |  |  | T2 | 3.57 (5.00) | 3.21 (4.43) |  |  | T2 | 8.73 (13.17) | 6.73 (10.32) |
|  | T3 | 4.07 (5.38) | 3.52 (5.55) |  |  | T3 | 3.35 (4.71) | 3.03 (4.56) |  |  | T3 | 8.64 (12.94) | 6.45 (10.06) |
|  | T4 | 4.15 (5.47) | 3.24 (5.05) |  |  | T4 | 3.51 (4.92) | 3.13 (4.86) |  |  | T4 | 9.13 (14.24) | 6.48 (10.78) |
| IL-4 | T0 | 81.86 (186.24) | 71.33 (194.74) |  | IL-13 | T0 | 17.01 (32.90) | 15.17 (36.44) |  | ITAC | T0 | 36.06 (17.68) | 31.62 (15.36) |
|  | T1 | 83.72 (183.14) | 73.88 (203.77) |  |  | T1 | 17.24 (32.95) | 14.47 (34.85) |  |  | T1 | 35.22 (18.30) | 30.12 (14.11) |
|  | T2 | 81.71 (178.10) | 76.40 (209.43) |  |  | T2 | 16.73 (31.39) | 14.92 (35.56) |  |  | T2 | 36.21 (17.07) | 31.30 (16.09) |
|  | T3 | 76.86 (177.03) | 70.93 (192.62) |  |  | T3 | 15.87 (30.79) | 14.43 (33.86) |  |  | T3 | 39.61 (30.32) | 29.53 (17.46) |
|  | T4 | 83.85 (187.51) | 73.36 (198.60) |  |  | T4 | 16.65 (31.60) | 14.66 (34.20) |  |  | T4 | 40.22 (26.09) | 28.73 (16.54) |
| IL-5 | T0 | 1.72 (2.45) | 1.51 (2.22) |  | IL-17A | T0 | 14.01 (21.17) | 14.31 (18.62) |  | MIP-1α | T0 | 85.06 (165.64) | 26.67 (32.68) |
|  | T1 | 1.80 (2.59) | 1.56 (2.36) |  |  | T1 | 14.77 (20.91) | 14.28 (18.07) |  |  | T1 | 94.21 (192.86) | 26.73 (32.20) |
|  | T2 | 1.91 (2.78) | 1.58 (2.35) |  |  | T2 | 15.16 (21.42) | 14.00 (17.79) |  |  | T2 | 81.22 (149.57) | 26.09 (31.74) |
|  | T3 | 1.73 (2.53) | 1.47 (2.20) |  |  | T3 | 15.59 (21.48) | 13.54 (18.30) |  |  | T3 | 90.76 (182.57) | 26.14 (34.09) |
|  | T4 | 1.86 (2.71) | 1.47 (2.24) |  |  | T4 | 15.75 (22.25) | 13.36 (18.72) |  |  | T4 | 91.04 (176.75) | 25.09 (32.59) |
| IL-6 | T0 | 3.90 (6.45) | 2.60 (4.27) |  | IL-21 | T0 | 6.88 (11.62) | 4.99 (8.22) |  | MIP-1β | T0 | 44.58 (47.42) | 16.83 (13.18) |
|  | T1 | 4.01 (6.39) | 2.71 (4.46) |  |  | T1 | 6.80 (11.67) | 5.10 (8.31) |  |  | T1 | 45.60 (49.41) | 16.55 (10.65) |
|  | T2 | 3.92 (6.10) | 2.87 (4.71) |  |  | T2 | 7.31 (12.45) | 4.94 (7.88) |  |  | T2 | 47.68 (49.51) | 16.41 (11.15) |
|  | T3 | 3.53 (5.41) | 2.76 (4.77) |  |  | T3 | 6.82 (11.33) | 5.28 (8.96) |  |  | T3 | 43.40 (45.11) | 15.52 (11.07) |
|  | T4 | 3.85 (6.01) | 2.79 (4.69) |  |  | T4 | 7.60 (12.95) | 5.14 (8.68) |  |  | T4 | 46.46 (47.88) | 13.96 (10.96) |
| IL-7 | T0 | 5.56 (5.01) | 3.52 (2.70) |  | IL-23 | T0 | 335.29 (476.73) | 216.81 (258.00) |  | MIP-3α | T0 | 39.02 (53.92) | 25.12 (36.04) |
|  | T1 | 5.57 (4.59) | 3.47 (2.56) |  |  | T1 | 346.74 (436.16) | 222.69 (257.20) |  |  | T1 | 59.76 (113.13) | 26.12 (36.80) |
|  | T2 | 5.76 (4.93) | 3.39 (2.45) |  |  | T2 | 351.44 (463.02) | 217.13 (245.74) |  |  | T2 | 48.35 (74.34) | 26.06 (37.95) |
|  | T3 | 5.86 (4.91) | 3.08 (2.55) |  |  | T3 | 315.75 (401.04) | 225.10 (295.48) |  |  | T3 | 39.55 (54.99) | 26.14 (37.23) |
|  | T4 | 5.73 (4.72) | 3.32 (2.71) |  |  | T4 | 347.85 (464.75) | 206.63 (268.56) |  |  | T4 | 40.27 (54.29) | 25.18 (38.56) |
| IL-8 | T0 | 17.14 (24.13) | 16.20 (36.84) |  | Fractalkine | T0 | 116.75 (143.76) | 81.35 (102.20) |  | TNF-α | T0 | 7.08 (5.86) | 5.12 (4.71) |
|  | T1 | 18.12 (24.49) | 14.99 (33.85) |  |  | T1 | 116.73 (134.91) | 82.17 (100.58) |  |  | T1 | 7.06 (5.40) | 4.95 (4.14) |
|  | T2 | 17.61 (23.65) | 15.10 (33.94) |  |  | T2 | 118.62 (142.05) | 83.02 (100.52) |  |  | T2 | 7.39 (5.67) | 5.11 (4.44) |
|  | T3 | 16.33 (22.06) | 14.86 (34.11) |  |  | T3 | 121.13 (145.44) | 79.53 (105.04) |  |  | T3 | 7.16 (5.68) | 5.13 (4.90) |
|  | T4 | 17.03 (23.20) | 15.68 (35.95) |  |  | T4 | 121.60 (143.73) | 78.71 (105.84) |  |  | T4 | 7.25 (5.52) | 4.81 (4.83) |

Cytokine concentrations are reported in pg/mL. Data are presented as mean (SD). IL, interleukin; GM-CSF, granulocyte-macrophage colony-stimulating factor; IFNγ, interferon gamma; ITAC, interferon-inducible T-cell alpha chemoattractant; MIP-1α, macrophage inflammatory protein-1 alpha; MIP-1β, macrophage inflammatory protein-1 beta; MIP-3α, macrophage inflammatory protein-3 alpha; TNF-α, tumor necrosis factor alpha.
